# Persistence of mRNA indicative of *Plasmodium falciparum* ring-stage parasites 42 days after artemisinin and non-artemisinin combination therapy in naturally infected Malians

**DOI:** 10.1186/s12936-020-03576-z

**Published:** 2021-01-09

**Authors:** Almahamoudou Mahamar, Kjerstin Lanke, Wouter Graumans, Halimatou Diawara, Koualy Sanogo, Kalifa Diarra, Sidi Mohamed Niambele, Roly Gosling, Chris Drakeley, Ingrid Chen, Alassane Dicko, Teun Bousema, Michelle E. Roh

**Affiliations:** 1 Malaria Research and Training Centre, Faculty of Pharmacy, Medicine, and Dentistry, University of Science, Techniques, and Technologies of Bamako, Bamako, Mali; 2 Radboud Institute for Health Sciences, Radboud University Medical Center, Geert Grooteplein Zuid 26-28, PO Box 9101, 6525GA Nijmegen, The Netherlands; 3 Global Health Group, Malaria Elimination Initiative, University of California, San Francisco, CA USA; 4 Department of Infection & Immunity, London School of Hygiene & Tropical Medicine, London, UK

**Keywords:** *Plasmodium falciparum*, Persistence, Artemisinin-based combination therapy, Dormancy, Recrudescence, Resistance, Gametocytes

## Abstract

**Background:**

Malaria control in sub-Saharan Africa relies upon prompt case management with artemisinin-based combination therapy (ACT). Ring-stage parasite mRNA, measured by *sbp1* quantitative reverse-transcriptase PCR (qRT-PCR), was previously reported to persist after ACT treatment and hypothesized to reflect temporary arrest of the growth of ring-stage parasites (dormancy) following exposure to artemisinins. Here, the persistence of ring-stage parasitaemia following ACT and non-ACT treatment was examined.

**Methods:**

Samples were used from naturally infected Malian gametocyte carriers who received dihydroartemisinin–piperaquine (DP) or sulfadoxine–pyrimethamine (SP–AQ) with or without gametocytocidal drugs. Gametocytes and ring-stage parasites were quantified by qRT-PCR during 42 days of follow-up.

**Results:**

At baseline, 89% (64/73) of participants had measurable ring-stage parasite mRNA. Following treatment, the proportion of ring-stage parasite-positive individuals and estimated densities declined for all four treatment groups. Ring-stage parasite prevalence and density was generally lower in arms that received DP compared to SP–AQ. This finding was most apparent days 1, 2, and 42 of follow-up (p < 0.01). Gametocytocidal drugs did not influence ring-stage parasite persistence. Ring-stage parasite density estimates on days 14 and 28 after initiation of treatment were higher among individuals who subsequently experienced recurrent parasitaemia compared to those who remained free of parasites until day 42 after initiation of treatment (p_day 14_ = 0.011 and p_day 28_ = 0.068). No association of ring-stage persistence with gametocyte carriage was observed.

**Conclusions:**

The current findings of lower ring-stage persistence after ACT without an effect of gametocytocidal partner drugs affirms the use of *sbp1* as ring-stage marker. Lower persistence of ring-stage mRNA after ACT treatment suggests the marker may not reflect dormant parasites whilst it was predictive of re-appearance of parasitaemia.

## Background

Malaria is a leading cause of global morbidity and mortality. In 2018, an estimated 228 million cases and 405,000 malaria-associated deaths were reported worldwide [1]. In sub-Saharan Africa, prompt diagnosis and treatment with artemisinin-based combination therapy (ACT) remains a key strategy for the treatment of uncomplicated *Plasmodium falciparum* malaria. Artemisinin-based combinations consist of an artemisinin derivative that rapidly reduces parasite burden and a partner drug with a longer half-life that clears remaining parasitaemia and provides prophylactic activity for weeks post-treatment. At present, artemisinin derivatives retain excellent efficacy in most of Africa despite decreased sensitivity to some of its partner drugs [2] and reports of emergence of artemisinin resistance in East Africa [3]. Whilst recent anti-malarial efficacy trials in Africa have shown overwhelmingly high treatment success after ACT (≥ 95%) [2], parasites may persist shortly after initiation of treatment [4]. Though this parasite persistence may not necessarily reflect drug resistance, which also depend on initial parasite density, host immunity, and drug absorption [5, 6], it is important to better understand what parasite populations persist and whether parasite persistence has consequences for later recrudescence [6].

Post-treatment detection of parasite DNA may reflect (remnants of) asexual parasites and gametocytes [7, 8], the latter commonly known to persist after ACT treatment [9]. A study in travellers in Sweden [10] indicated that residual parasite DNA can be detected by qPCR for up to 42 days after successful treatment without evidence of viable asexual parasites or gametocytes. Recently, mRNA transcripts specific to ring-stage parasites (skeleton binding protein; *sbp1*) were reported following ACT treatment [7, 11]. This apparent persistence of low-level asexual parasitaemia after ACT may be explained by the “dormancy theory” [12] which postulates that under artemisinin pressure, a subpopulation of young ring-stage parasites undergo developmental arrest where they remain metabolically inactive. It has been suggested that these low-density ring-stage parasites may represent ‘sleeping beauties’ (i.e. dormant parasites that can tolerate artemisinin treatment, but are sensitive to other anti-malarials) [13, 14] and this mechanism may explain why certain individuals experience recrudescence in the absence of actual artemisinin resistance. Recent evidence from controlled infections with artemisinin-sensitive *Plasmodium falciparum* 3D7 parasites suggests that dormant parasites can be induced by artemisinin monotherapy and provides a plausible explanation for recrudescences [15]. Published in vitro evidence [7], demonstrates that these dormant parasites are expected to recover continuously over 25 days causing recrudescence.

The aim of this study was to expand on earlier observations by examining ring-stage parasitaemia among trial participants that were followed for 42 days after being randomized to ACT or non-ACT anti-malarials with and without gametocytocidal drugs. This study allowed examination whether persisting ring-stage parasitaemia persists after administration of non-ACT, and whether the persistence of ring-stage parasites is associated with parasite recrudescence and/or continued gametocyte production.

## Methods

### Ethics statement

Ethical approval for the study was granted by the Ethics Committee of the Faculty of Medicine, Pharmacy, and Dentistry of the University of Science, Techniques, and Technologies of Bamako (Bamako, Mali), the Committee on Human Research at the University of California San Francisco (UCSF; San Francisco, CA, USA), and the Research Ethics Committee of the London School of Hygiene & Tropical Medicine (London, UK).

### Study cohort and sample collection

This study used samples obtained from participants of a trial in Ouélessébougou, Mali who were randomized 1:1:1:1 to receive either sulfadoxine–pyrimethamine (SP–AQ), SP–AQ with single low-dose primaquine (SP–AQ + PQ), dihydroartemisinin-piperaquine (DP), or dihydroartemisinin–piperaquine with methylene blue (DP + MB) [16]. Eligible participants were males with asymptomatic *P. falciparum* mono-infection, between 5 and 50 years of age, who were glucose-6-phosphate dehydrogenase (G6PD)-normal by CareStart G6PD rapid diagnostic test (Access Bio, Somerset, NJ, USA), had a hemoglobin concentration of ≥ 10 g/dL, and had a *P. falciparum* gametocyte density of ≥ 2 gametocytes/500 white blood cells by thick film microscopy. Participants were excluded if they had a serious or chronic illness (including signs of severe malaria), weighed 80 kg or more, reported anti-malarial use within 7 days of screening (artemether–lumefantrine and artesunate–amodiaquine being first-line treatments), or reported allergies to study drugs.

Details on the study procedures are described in the original paper [16]. In brief, participants were followed for 42 days and blood samples were obtained on days 0, 1, 2, 7, 14, 28 and 42 after initiation of treatment. Blood smear microscopy was conducted on blood samples taken by finger prick on day 0 and all days of follow-up to assess for asexual parasite and gametocyte density. For the measurement of ring-stage and gametocyte density by qRT-PCR, 100 μL of blood was collected in EDTA tubes and immediately transferred to RNAprotect (Qiagen) and stored at − 80 °C until extraction by MagNAPure LC automated extractor (Total Nucleic Acid Isolation Kit-High Performance; Roche Applied Science, Indianapolis, IN, USA).

### Laboratory analysis

Ring-stage parasites were quantified by qRT-PCR targeting the *sbp1* mRNA transcript using previously described methods [7]. The limit of quantification of this assay is in the range of 1 parasite/µL[7], the limit of detection is 0.01 parasite/µL Male and female gametocyte densities were quantified by qRT-PCR, targeting male *PfMGET* and female *Pfs25* mRNA transcripts as described elsewhere [16]. For samples that were microscopy-positive for asexual *P. falciparum* parasites on days 7, 14, 28, and 42, PCR genotyping of *glurp* (glurp2), *msp2* (Fc27), *msp1* (K1, MAD20 and RO33) and *lc1* alleles were performed on samples obtained at enrollment and day of post-treatment failure. Pre- and post-treatment pairs were analysed and classified as either recrudescent, re-infection, or indeterminate infections according to World Health Organization guidelines [8, 17].

### Sample size considerations

The sample size for this study was dictated by the original clinical trial, with 20 individuals per study arm to allow a within-person change in infectivity after treatment [16]. It is acknowledged that this sample size is insufficient to quantify with precision the proportion of individuals with persisting ring-stage parasites post treatment. Despite modest in sample size, the current study samples do allow, a unique comparison of parasite persistence following ACT and non-ACT treatment. The latter group (i.e. individuals with confirmed parasite carriage receiving non-ACT treatment) is rare among clinical trials and allows exploration of the hypothesis that ring-stage parasite persistence is specific for ACT treatment [12–14].

### Statistical analysis

All analyses were performed using Stata 14.0 (StataCorp, College Station, TX, USA) and R (version 3.5.0; R Project for Statistical Computing; http://www.r-project.org/). Comparisons between proportions were conducted using Chi-squared or Fisher’s exact test and Mann–Whitney tests were used to compare differences in parasite densities, unless otherwise specified. Correlations between ring-stage and gametocyte parasite densities were assessed by Spearman’s rank correlation coefficient using the log_10_ transformed versions of these variables. Generalized estimating equations were used to compare SBP-1 parasite prevalence and density between SP–AQ and DP arms, accounting for repeated observations for individuals. Log-binomial regression was used to model relative risk ratios and linear regression was used to model mean differences in log10-transformed SBP-1 parasite density. Models adjusted for baseline log10-transformed SBP-1 parasite density. An interaction term between treatment and follow-up visit was included to assess whether participants of the DP group cleared parasitaemia at a more rapid rate than SP–AQ. An overall F-test was used compute the p-value testing joint effect of the interaction terms. Log-binomial regression models were used to assess whether age, weight, treatment arm, or baseline SBP-1 parasite density were associated with persistent parasitaemia on day 7 post-treatment. All tests were two-sided with alpha = 0.05. p-values < 0.05 were considered statistically significant.

## Results

### Characteristics of sample population

The study included 73/80 (91%) males from the original trial [16] (Table 1). Seven participants withdrew consent. Of the 73 remaining, 75% were between 5 and 14 years old and randomized to receive SP–AQ (n = 18), SP–AQ + PQ (n = 18), DP (n = 18); or DP + MB (n = 19). In this population, recruited based on microscopy-detectable gametocyte carriage, 64 (89%) had a measurable density of ring-stage parasites by qRT-PCR. This indicates that a minority were individuals who had gametocytes only and cleared their initial asexual infection. At baseline, ring-stage parasite density by qRT-PCR was positively correlated with microscopy-detected asexual parasite density (Spearman’s rho = 0.83, p < 0.0001). Younger participants (between 5 and 14) had higher baseline ring-stage parasite densities compared to those 15 years and above (p < 0.02). Baseline ring-stage parasite density estimates were similar between randomized treatment groups (p = 0.61).

**Table 1:** Characteristics of study population

Baseline characteristics	All (n = 73)
Age group in years, n (%)	
5 to < 15	55 (75)
15 to < 25	12 (16)
25+	6 (8)
Weight in kg (mean ± SD)	33.4 ± 15.2
Treatment, n (%)	
SP–AQ	18 (25)
SP–AQ + PQ	18 (25)
DP	18 (25)
DP + MB	19 (26)
Asexual parasite prevalence by microscopy, n (%)	43 (59)
Asexual parasite density by microscopy (per μL), median [IQR]	107 [0, 400]
Ring-stage parasite prevalence, n (%)	64 (89)
Ring-stage parasite density (per μL), median [IQR]	633 [7, 2028]
Gametocyte prevalence by qRT-PCR, n (%)	72 (100)
Gametocyte density by qRT-PCR (per μL), median [IQR]	57 [32, 173]

Values may not up to 100% due to missing values

*IQR* interquartile range

### Parasite kinetics following treatment

**Fig. 1:**
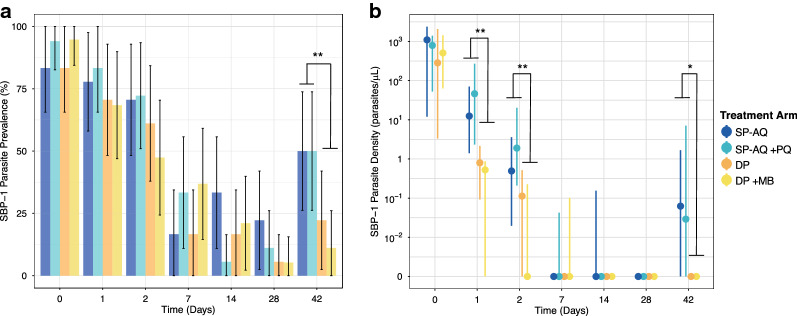
SBP-1 parasite prevalence (**a**) and density (**b**) between treatment arms over time. p-values were calculated using chi-squared or Fisher’s exact test to test differences in prevalence between ACT and non-ACT groups and Wilcoxon’s rank-sum test to test differences in parasite density. **p-value < 0·0001, *p < 0.05

Following anti-malarial treatment, the prevalence and density of ring-stage parasites reduced across all four treatment arms (Fig. 1; Tables 2, 3). Ring-stage kinetics were similar between SP–AQ and SP–AQ + PQ arms and between DP vs DP + MB arms (Fig. 1; Additional file 1: Tables S1, S2), prompting us to combine arms receiving SP–AQ and arms receiving DP. Ring-stage parasite prevalence was, on average, 24% lower in the combined DP group compared to the combined SP–AQ group (RR_overall_ = 0.76 [95% CI 0.62, 0.94]; p = 0.012) (Table 2). Similarly, estimated SBP-1 parasite density was, on average, 82% [95% CI 62, 92] lower in combined DP group compared to the SP–AQ group (p < 0.001). This finding was most apparent days 1, 2, and 42 of follow-up (p < 0.01) (Table 3). Models including an interaction term between DP and follow-up visit found DP was associated with a more rapid reduction in ring-stage parasite density than SP–AQ (p_DP × time_ = 0.005).

**Table 2:** Comparison of qRT-PCR SBP-1 prevalence between SP–AQ and DP treatment arms over time

Visit	SBP-1 qRT-PCR					Microscopy				
	Prevalence, n (%)			DP: SP–AQ PR [95% CI]	p-value^1^	Prevalence, n (%)			DP: SP–AQ PR [95% CI]	p-value^1^
	All	SP–AQ	DP			All	SP–AQ	DP DP		
Day 0	64 (89)	31 (89)	33 (89)	1.01 [0.86, 1.19]	0.93	43 (59)	24 (67)	19 (51)	0.77 [0.52, 1.14]	0.18
Day 1	54 (75)	29 (81)	25 (69)	0.86 [0.66, 1.13]	0.28	19 (26)	16 (44)	3 (8)	0.18 [0.05, 0.57]	0.0004
Day 2	45 (63)	25 (71)	20 (54)	0.76 [0.53, 1.09]	0.13	9 (12)	6 (17)	3 (8)	0.49 [0.13, 1.80]	0.27
Day 7	19 (26)	9 (25)	10 (27)	1.08 [0.50, 2.35]	0.84	1 (1)	0 (0)	1 (3)	–	–
Day 14	14 (19)	7 (19)	7 (19)	0.98 [0.38, 2.50]	0.96	1 (1)	1 (3)	0 (0)	–	–
Day 28	8 (11)	6 (17)	2 (5)	0.32 [0.07, 1.50]	0.15	1 (1)	1 (3)	0 (0)	–	–
Day 42	24 (33)	18 (50)	6 (17)	0.33 [0.15, 0.74]	0.0027	7 (11)	4 (11)	3 (8)	0.75 [0.18, 3.11]	0.69

*PR* prevalence ratio, *SP–AQ* sulfadoxine–pyrimethamine and amodiaquine, *DP* dihydroartemisinin–piperaquine

^1^ p-values computed using Chi-squared test or Fisher’s exact test if any cell value in 2 × 2 table was less than five

**Table 3:** Comparison of SBP-1 parasite density between DP and SP-AQ arms

Visit	Median density (per μL) [IQR]		Difference in log_10_ SBP-1 Density		
	SP-AQ	DP	DP:SP-AQ MD [95% CI]^1^	DP:SP-AQ % Difference [95% CI]^2^	p-value
Day 0	891.3 [8.2, 2065.4]	330.4 [6.5, 1990.7]	0.01 [− 1.0, 1.0]	3 [− 91, 1019]	0.98
Day 1	21.2 [0.6, 212]	0.6 [0, 2.3]	− 1.5 [− 2.0, − 0.9]	− 97 [− 99, − 89]	< 0.001
Day 2	0.6 [0, 11.2]	0.02 [0, 0.3]	− 1.2 [− 1.8, − 0.6]	− 94 [− 98, 76]	< 0.001
Day 7	0 [0, 0.003]	0 [0, 0.07]	0.09 [− 0.4, 0.6]	24 [− 64, 320]	0.73
Day 14	0 [0, 0]	0 [0, 0]	− 0.3 [− 0.8, 0.3]	− 44 [− 84, 96]	0.36
Day 28	0 [0, 0]	0 [0, 0]	− 0.4 [− 0.8, 0.08]	− 57 [− 85, 19]	0.10
Day 42	0.03 [0, 7.1]	0 [0, 0]	− 1.2 [− 2.1, − 0.3]	− 94 [− 99, − 46]	0.012

*IQR* interquartile range

^1^ Coefficient from linear regression modelling log10 SBP-1 parasite density. Models comparing parasite density at non-baseline visits adjusted for baseline SBP-1 values. A value of 0.001 was added to all values to account for zeroes

^2^ Calculated by 100 * (10^MD^ − 1)

Despite anti-malarial treatment, ring-stage parasite RNA was still detectable across all days of follow-up and all treatment arms (Table 2). On day 7 post-treatment, 14 (19%) participants had detectable levels of ring-stage parasitaemia, despite only one person being microscopy-positive for asexual parasites (who was later classified as having a recrudescent infection). Multivariate log-binomial regression was used to determine which factors were associated with the presence of ring-stage parasites on day 7. Participants with persistent parasitaemia on day 7 were more likely to have higher ring-stage parasite densities at baseline (relative risk ratio = 1.31 [95 CI 1.00, 1.73] increase in risk for every 1% increase in log10-transformed baseline SBP1 parasite density; p = 0.049). However, age (p = 0.84), weight (p = 0.92), and treatment type (p = 0.30) were not statistically significant predictors of persistent parasitaemia on day 7.

**Fig. 2:**
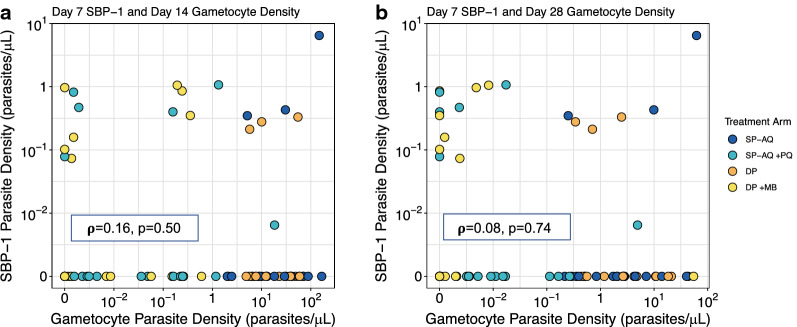
Correlation between Day 7 SBP-1 Parasite Density and Gametocyte Density on Days 14 (**a**) and 28 (**b**). Correlation (ρ) and p-values were calculated only among those who had detectable levels of ring-stage parasites on day 7 using Spearman’s rank correlation coefficient

Spearman rank correlation tests were used to assess whether the persistence of ring-stage parasites was associated with later gametocyte density, which would be indicative of ongoing gametocyte production. Among those who had persistent ring-stage parasitaemia on day 7 (n = 14), no significant correlation was observed between their ring-stage parasite density on day 7 and gametocyte density on days 14 and 28 (Fig. 2).

### Factors associated with recurrent infections

**Fig. 3:**
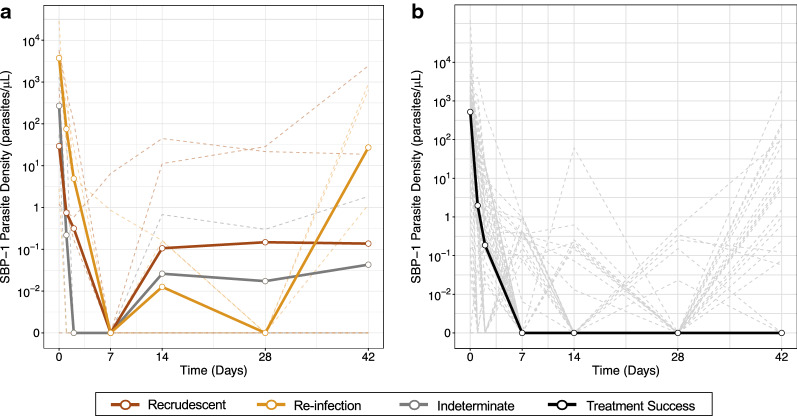
SBP-1 Parasite Density by Recurrent Infections and Days of Follow-up. **a** Includes participants with either recrudescent (dark red), re-infection (orange), or indeterminate (dark grey) infections. **b** Includes only participants who were successfully treated. Dark, bold lines indicate median values at each time point. Light, dashed lines indicate individual trajectories

Over the course of the 42-day follow-up, 10/73 (14%) participants experienced recurrent parasitaemia detectable by microscopy (4, 4 and 2 in the DP + MB, SP–AQ and SP–AQ  + PQ arm, respectively). Conventional genotyping of polymorphic MSP-1, MSP-2 and GLURP genes [17] indicated that four of these were recrudescent infections, four were re-infections, and two were indeterminate (Additional file 1: Table S3). Seven of the recurrent infections were detected on day 42 (2 indeterminate, 2 recrudescent, and 3 reinfections) and the rest occurred on days 7 (1 recrudescent), 14 (1 recrudescent), and day 28 (1 reinfection) (Table 4). SP–AQ was associated with a higher risk of recurrent infection compared to DP, though this finding did not reach statistical significance (RR for DP = 0.63 [95% CI 0.19, 2.03]; p = 0.44). Ring-stage parasite densities on days 14 and 28 after treatment initiation were higher among individuals who subsequently experienced recurrent parasitaemia compared to those who did not experience recurrent infection until day 42 after initiation of treatment (Fig. 3) (Mann Whitney test p_day 14_ = 0.011 and p_day 28_ = 0.068) (Table 4).

**Table 4:** Prevalence and density of ring-stage parasites by recurrent infection type

Infection Type	Prevalence, n (%)	Ring-stage parasite density (per μL), median [IQR]					
		Day 7	p-value	Day 14	p-value	Day 28	p-value
Treatment success	63 (86)	0 [0, 0.07]	Ref	0 [0, 0]	Ref	0 [0, 0]	Ref
Recurrent	10 (14)	0 [0, 0]	0.99	0.08 [0, 0.4]	0.011	0 [0, 0.3]	0.068
Recrudescent	4 (5)	0 [0, 6.5]	0.57	5.6 [0,11.2]	0.12	14.2 [0, 28.4]	0.029
Re-infection	4 (5)	0 [0, 0.4]	0.92	0.08 [0, 0.2]	0.085	0 [0, 0]	0.61
Indeterminate	2 (3)	0 [0, 0]	0.40	0.3 [0, 0.7]	0.12	0.1 [0, 0.3]	0.041

To assess whether prior ring-stage parasite densities were associated with increased risk of recurrent infections, parasite densities for each column of recurrent infections excludes those that were detected before or on that day of follow-up. For example, ring-stage parasite densities for recrudescent infections on day 14 excludes recrudescent infections that occurred on day 7 (n = 1) and day 14 (n = 1)

p-values were computed using the Mann–Whitney test. Comparisons are between recurrent infection categories to reference category (Treatment Success)

## Discussion

In this study of young Malian males with asymptomatic *P. falciparum* carriage, ring-stage parasite mRNA was detected up to 42 days after anti-malarial treatment. Estimated densities of post-treatment ring-stage parasites reduced at a more rapid rate following receipt of ACT compared to non-ACT, arguing against the hypothesis that this signal reflects dormant parasites that tolerate can artemisinin treatment. This could, however, also be obscured by differences in treatment efficacy between DP and SP–AQ. In this modestly sized population, few individuals experienced an episode of recurrent parasitaemia (n = 10), but these individuals tended to harbour higher ring-stage parasite densities prior to recurrence than those who were successfully treated.

Whilst repeated assessments of parasite density shortly after initiation of treatment provide the most conclusive evidence on (changes in) parasite responsiveness [18], alternative metrics are used to compare the early effects of anti-malarials. These include the proportion of individuals with residual parasitaemia by microscopy [19] or PCR [4, 8] or the concentration of the histidine rich protein-2 parasite antigen [20]. The current study, examining the kinetics of mRNA transcripts indicative of ring-stage parasitaemia following treatment [11, 21], explicitly does not aim to present this measure as a proxy for parasite clearance half-lives or evidence of reduced susceptibility of parasites to treatment. A recent study from Mali that was specifically designed to assess parasite clearance half-lives following artesunate monotherapy observed indications for delayed clearance in one setting [4], highlighting the need for monitoring of (early) parasite clearance following ACT. Here, the aim was to examine a previously reported phenomenon of persisting *sbp1* ring-stage transcripts following ACT treatment in more detail [7, 11]. Previous studies reported weak [7, 11] or absent association with the concurrent presence of gametocytes [21]. Along with in vitro experiments on synchronized parasite material [11], this make a strong case that this marker is indeed specific to the detection of ring-stage parasites. The current data further support this by reporting no measurable impact of gametocytocidal drugs on ring-stage mRNA persistence. It was hypothesized that ring-stage parasites post ACT treatment are reflective of parasite dormancy, a phenomenon specific to artemisinin derivatives [14] where parasites are able to tolerate artemisinin treatment by entering a temporary growth-arrested state. Previous in vitro work indicated that parasites that became dormant after a single treatment with dihydroartemisinin were still receptive to other drugs [14]. The study, directly comparing ACT and non-ACT treatment, allowed us to test this hypothesis. Ring-stage parasites were present across all treatment arms and at higher prevalence and density following non-ACT treatment. Whilst sample size is limited, it would argue against the artemisinin-specific dormancy phenomenon as (only) explanation for ring-stage persistence.

From a public health perspective, it is important to examine whether persisting ring-stage parasites are predictors of recrudescent infections or the source of gametocyte production. The current study population was small and as the original study objective was to assess gametocyte clearance and infectivity [16], not all individuals harbored asexual parasites at the start of treatment. Only 10 episodes of recurrent parasitaemia were observed and only four of these represented recrudescent infections by conventional parasite genotyping. Nevertheless, ring-stage parasitaemia appeared higher prior to the occurrence of recurrent infection. Interestingly, ring-stage persistence was not only higher among recrudescent infections but also among participants who later experienced apparent re-infections or recurrent parasitaemia that was not classifiable as either recrudescent or re-infection. Whether this reflects imperfect genotyping, with recrudescent infections being misclassified, or early development of re-infections in a phase when effective prophylaxis is expected [22], is unclear.

The longer period of follow-up in this study also allowed the exploration of associations of ring-stage persistence with subsequent gametocyte carriage. Whilst data collection was not specifically designed for this, it was possible to relate ring-stage densities with gametocyte densities 7 and 14 days later, roughly the period needed for gametocyte production [23], and observed no association. The investment of parasites that persist under drug-pressure in either asexual multiplication or gametocyte production reflects a delicate balance [24]. A terminal investment in gametocyte production, sometimes hypothesized when increased gametocyte production is seen in partially resistant parasites [25, 26], was not observed here.

The study is subject to several limitations. First, it is recognized that sample size was small, which may have limited the statistical power of the study. Thus, future studies (e.g. pooled analyses) may be needed to confirm findings. Second, the study population consisted mostly of young males with high gametocyte densities at enrollment, which may limit the generalizability of findings to other parasitized populations. The study population is not representative of individuals with uncomplicated malaria; the average duration of infection will have been longer for the current study population who all had blood-stage infections sufficiently long to complete the 8–12 day maturation period of gametocytes [27]. This will have affected parasite stage composition at presentation, reflected by the fact that a minority of individuals did not have detectable asexual parasites at enrolment. Third, whilst three targets were included for genotyping, only a single baseline sample was compared with a single day of recurrent parasitaemia. Sampling over multiple days after enrolment may have increased the detectability of circulating parasite strains [8, 28]. It is, therefore, conceivable that more of the recurrent infections are in fact already present prior to treatment and could thus be classified as recrudescent infections. Fourth, due to the uneven follow-up periods and longer tailed follow-up periods toward end of the study, it was not possible to assess the exact time of recurrent infection and how long ring-stage parasitaemia remains elevated prior to recurrence. Fifth, the markedly longer parasite clearance time for SP–AQ arm may reflect resistance of parasite populations to this drug combination, making it difficult to compare the impact of the artemisinin component on ring-stage persistence. Even longer follow-up periods may have uncovered whether the apparent rise in ring-stage parasite prevalence and density towards the end of the study period may have resulted in more recrudescent infections. The last important limitation, similar to previous studies, is that the current study provides to definitive evidence that mRNA reflects viable parasites. This would require post-treatment cultures [15].

## Conclusion

In summary, the current findings indicate that ring-stage parasites may persist at low concentrations following anti-malarial treatment. Whilst this parasite population is unlikely to reflect dormant parasites following artemisinin treatment, the association of ring-stage persistence with subsequent detection of recurrent parasitaemia by microscopy warrants further studies to examine whether they may reflect viable parasite populations that may recrudesce when the concentrations of anti-malarials become permissive during follow-up.

## Supplementary Information

**Additional file 1: Table S1.** Prevalence of SBP-1 on days of follow-up by treatment arm. **Table S2.** SBP-1 Parasite density on days of follow-up by treatment arm. **Table S3.** Recrudescence-reinfection genotyping.

## Data Availability

The datasets used in the current study are available from the corresponding author on reasonable request.

## Abbreviations

ACT: Artemisinin-based combination therapy
DP: Dihydroartemisinin–piperaquine
G6PD: Glucose-6-phosphate dehydrogenase
MB: Methylene blue
PQ: Primaquine
qRT-PCR: Real-time reverse transcriptase polymerase chain reaction
SBP1: Skeleton binding protein 1
SP–AQ: Sulfadoxine–pyrimethamine
