# Supplementary material for: Persistence of mRNA indicative of Plasmodium falciparum ring-stage parasites 42 days after artemisinin and non-artemisinin combination therapy in naturally infected Malians
Source: Malar J. 2021 Jan 9;20:34. doi: 10.1186/s12936-020-03576-z (PMC7797096; doi:10.1186/s12936-020-03576-z)
Supplement: Supplementary file 1 — Additional file 1: Table S1. Prevalence of SBP-1 on days of follow-up by treatment arm. Table S2. SBP-1 Parasite density on days of follow-up by treatment arm. Table S3. Recrudescence-reinfection genotyping. [file 12936_2020_3576_MOESM1_ESM.docx]

**Table S1.** Prevalence of SBP-1 on days of follow-up by treatment arm.

| **Visit** | **Prevalence, n/N (%)** | | | |  | **p-value_SP-AQ vs DP_^1^** | **p-value_gametocytocidal_^2^** |
| --- | --- | --- | --- | --- | --- | --- | --- |
|  | **SP-AQ** | **SP-AQ +PQ** | **DP** | **DP +MB** |  |  |  |
| 0 | 15/18 (83) | 16/17 (94) | 15/18 (83) | 18/19 (95) |  | 0.93 | 0.13 |
| 1 | 14/18 (78) | 15/18 (83) | 12/17 (71) | 13/19 (68) |  | 0.28 | 0.89 |
| 2 | 12/17 (71) | 13/18 (72) | 11/18 (61) | 9/19 (47) |  | 0.13 | 0.58 |
| 7 | 3/18 (17) | 6/18 (33) | 3/17 (17) | 7/19 (36) |  | 0.84 | 0.07 |
| 14 | 6/18 (33) | 1/18 (6) | 3/18 (17) | 4/19 (21) |  | 0.96 | 0.25 |
| 28 | 4/18 (22) | 2/18 (11) | 1/18 (6) | 1/19 (5) |  | 0.15 | 0.48 |
| 42 | 9/18 (50) | 9/18 (50) | 4/18 (22) | 2/18 (11) |  | 0.0027 | 0.62 |

Abbreviations: DP=dihydroartemisinin-piperaquine; MB=methylene blue; PQ=primaquine; SP-AQ=sulfadoxine-pyrimethamine and amodiaquine

^1^ Reported p-values from Fisher’s exact test comparing SP-AQ and DP arms (i.e. combined SP-AQ and SP-AQ+PQ arms versus combined DP and DP+MB arms)

^2^ Reported p-values from Fisher’s exact test comparing arms that received gametocytocidal drugs versus those that did not (i.e. combined SP-AQ+PQ and DP+MB arms versus combined SP-AQ and DP arms)

**Table S2.** SBP-1 Parasite density on days of follow-up by treatment arm

| **Visit** | **SBP-1 Parasite Density (per μL), median [IQR]** | | | |  | **p-value_SP-AQ vs DP_^1^** | **p-value_gametocytocidal_^2^** |
| --- | --- | --- | --- | --- | --- | --- | --- |
|  | **SP-AQ** | **SP-AQ +PQ** | **DP** | **DP +MB** |  |  |  |
| 0 | 1097 [3, 3054] | 793 [49, 1416] | 284 [0.1, 2404] | 508 [55, 1959] |  | 0.98 | 0.28 |
| 1 | 12 [1, 95] | 46 [1, 310] | 0.8 [0, 2] | 0.5 [0, 2] |  | <0.001 | 0.56 |
| 2 | 0.5 [0, 5] | 2 [0, 22] | 0.1 [0, 1] | 0 [0, 0.3] |  | <0.001 | 0.68 |
| 7 | 0 [0, 0] | 0 [0. 0.1] | 0 [0,0] | 0 [0, 0.2] |  | 0.73 | 0.27 |
| 14 | 0 [0, 0.2] | 0 [0, 0] | 0 [0,0] | 0 [0, 0] |  | 0.36 | 0.25 |
| 28 | 0 [0, 0] | 0 [0, 0] | 0 [0,0] | 0 [0, 0] |  | 0.10 | 0.21 |
| 42 | - 1. [0, 2] | 0.03 [0, 8] | 0 [0,0] | 0 [0, 0] |  | 0.012 | 0.73 |

Abbreviations: DP=dihydroartemisinin-piperaquine; IQR=interquartile range; MB=methylene blue; PQ=primaquine; SP-AQ=sulfadoxine-pyrimethamine and amodiaquine

^1^ Reported p-values from comparing SP-AQ and DP arms (i.e. combined SP-AQ and SP-AQ+PQ arms versus combined DP and DP+MB arms). P-values computed from linear regression modelling log10 SBP-1 parasite density. Models comparing parasite density at non-baseline visits adjusted for baseline SBP-1 values. A value of 0.001 was added to all values to account for zeroes.

^2^ Reported p-values from comparing arms that received gametocytocidal drugs versus those that did not (i.e. combined SP-AQ+PQ and DP+MB arms versus combined SP-AQ and DP arms). P-values were computed similarly to those comparing SP-AQ and DP arms.

**Table S3.** Recrudescence-reinfection genotyping. Results of genotyping for 3 polymorphic markers, using WHO criteria for defining recrudescent infections [1] A ‘new infection’ is a subsequenst occurring parasitaemia in which all the alleles in parasites from the post-treatment sample are different from those in the admission sample, for one or more loci tested. In a ‘recrudescence’, at least one allele at each locus is common to both paired samples. Indeterminate where those samples where results were only available for one locus.

|  | **GLURP** | **MSP2** | **MSP1** |  |  |  |  |
| --- | --- | --- | --- | --- | --- | --- | --- |
|  | **GLURP** | **Ic1** | **R033** | **K1** | **Fc27** | **Mad20** | **outcome** |
| PQ03-02 D0 | 700-800 | 480 | - | 200 | 290 | - | reinfection |
| PQ03-02 D42 | 900+1250 | 500+520 | - | 180+230+350 | 310 | 200 |  |
| PQ03-20 D0 | 800 | 500 | 150 | 200+230+350 | - | - | indeterminate |
| PQ03-20 D42 | - | - | - | 180+230+350 | - | - |  |
| PQ03-42 D0 | 520+700+780+900+950+1000+1050 | 480+580 | 150 | 200+230+330 | 290 | 180 | recrudescence |
| PQ03-42 D14 | 700+780+900+950+1000 | 480+580 | - | 180+230+270+330 | - | - |  |
| PQ03-08 D0 | 780 | 480 | - | 200+350 | - | - | recrudescence |
| PQ03-08 D7 | 780 | - | - | 200+350 | - | - |  |
| PQ03-24 D0 | 980 | 480 | - | 230+370+400 | - | - | reinfection |
| PQ03-24 D42 | 980 | 480 | - | 200+320 | - | - |  |
| PQ03-59 D0 | 590+900 | 480+580 | - | 250 | 290 | 200 | reinfection |
| PQ03-59 D42 | - | - | - | 370+400 | - | - |  |
| PQ03-18 D0 | 900 | 480 | - | 200+370 | - | - | recrudescence |
| PQ03-18 D42 | - | 480 | - | 180+230+370+400 | - | - |  |
| PQ03-33 D0 | 900+950+980 | 480+500+580 | 150 | 230+250+350 | 270+290+310+330 | - | recrudescence |
| PQ03-33 D42 | 680+780+900+950+980 | 480+550 | 150 | 180+250+350 | 330 | 180+220 |  |
| PQ03-79 D0 | 650+680+720+800+870+900 | 480+580 | 150 | 230+250+350 | 270 | - | reinfection |
| PQ03-79 D28 | 680+800+870+900 | - | - | 180+370 | - | - |  |
| PQ03-19 D0 | 900 | 480+580 | - | 180+230+250+370 | - | - | indeterminate |
| PQ03-19 D42 | - | - | - | 370 | - | - |  |

1. World Health Organization: *Methods and techniques for clinical trials on antimalarial drug efficacy: genotyping to identify parasite populations: informal consultation organized by the Medicines for Malaria Venture and cosponsored by the World Health Organization, 29-31 May 2007, Amsterdam, The Netherlands.* World Health Organization; 2008.
